# A deep learning framework for comprehensive prediction of human RNA G-quadruplex-binding proteins

**DOI:** 10.1093/bioinformatics/btag088

**Published:** 2026-02-19

**Authors:** Serena Rosignoli, Sophie Taraglio, Francesco Di Luzio, Elisa Lustrino, Dario Marzella, Arne Elofsson, Massimo Panella, Alessandro Paiardini

**Affiliations:** Centre for Regenerative Medicine “Stefano Ferrari”, Department of Life Sciences, University of Modena and Reggio Emilia, Modena 41125, Italy; Department of Biochemical Sciences “A. Rossi Fanelli”, Sapienza University of Rome, Rome 00185, Italy; Department of Information Engineering, Electronics and Telecommunications, Sapienza University of Rome, Rome 00184, Italy; Department of Biochemical Sciences “A. Rossi Fanelli”, Sapienza University of Rome, Rome 00185, Italy; Medical BioSciences Department, Radboud University Medical Center, Nijmegen 6500, The Netherlands; Department of Biochemistry and Biophysics and Science for Life Laboratory, Stockholm University, Solna 171 21, Sweden; Department of Information Engineering, Electronics and Telecommunications, Sapienza University of Rome, Rome 00184, Italy; Department of Biochemical Sciences “A. Rossi Fanelli”, Sapienza University of Rome, Rome 00185, Italy

## Abstract

**Motivation:**

G-quadruplex-binding proteins (G4BPs) play key roles in RNA metabolism and stress response, yet their identification remains experimentally challenging. Here, we present a deep learning (DL) framework for the prediction of RNA G4BPs (RG4BPs), integrating diverse encoding strategies and neural architectures. Our best-performing model, which includes ESM-2 protein language model embeddings and consists of an LSTM architecture, achieved 86% accuracy in distinguishing RG4BPs from non-binder proteins. The application of this model to the human proteome uncovered 2160 high-confidence RG4BP candidates, many of which display intrinsically disordered regions (IDRs) and enrichment in stress granule organelles. These findings reveal a potential link between G-quadruplex recognition and cellular stress responses. To enable easy and broad access to the framework, we developed G^4^REP, a web server for RG4BP prediction and analysis. Overall, an effective approach to explore the RG4BPs landscape and uncover novel players in RNA regulation is provided.

**Availability:**

Source code for the *G^4^REP Model* training and evaluation is available at: https://github.com/G4REP/G4REPmodel and at https://doi.org/10.5281/zenodo.17963046. *G^4^REP Server* is hosted at: https://schubert.bio.uniroma1.it/g4/

## Graphical Abstract

**Graphical Abstract:**
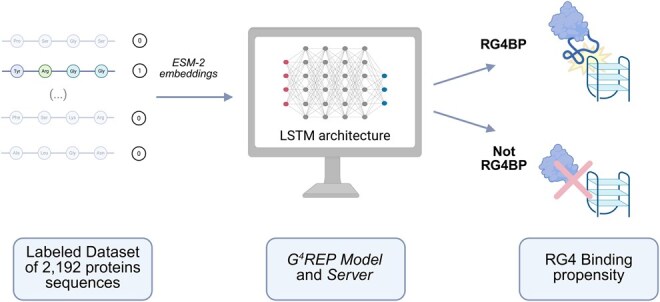

## 1 Introduction

RNA G-quadruplexes (RG4s), formed by guanine-rich sequences that fold into a four-stranded architecture, have recently gained significant attention due to their involvement in essential cellular processes (Malgowska *et al.* 2016, Yang 2019, Diaz Escarcega *et al.* 2024). RG4s are found in both protein-coding and non-coding transcripts, where they play roles in diverse aspects of RNA functionality, such as mRNA transport, maturation, degradation, splicing, microRNA regulation, PIWI-interacting RNA generation, stress response, and ribosomal RNA reorganization (Fay *et al.* 2017, Diaz Escarcega *et al.* 2024). While the precise biological roles of RNA G4s remain under investigation, emerging research highlights their dynamic nature and regulatory impact on gene expression (Fay *et al.* 2017, Dumas *et al.* 2021). These structures also influence cellular compartmentalization and stress responses, making them potential targets for therapeutic applications (Dumas *et al.* 2021, Kharel *et al.* 2024). In this regard, RG4 structures have been identified in stress granules (SG), membraneless organelles formed during the cytoplasmic aggregation of proteins and RNAs in response to cellular stress (Turner *et al.* 2022), and SG-associated proteins have been demonstrated to directly and selectively bind RG4s (He *et al.* 2021), reinforcing their functional relevance in stress adaptation. Proteins able to recognize and bind G4s are referred to as G4 binding proteins (G4BPs) and are classified into DNA or RNA G4BPs (DG4BPs and RG4BPs, respectively) (Shu *et al.* 2022). The interaction between RG4s and their binding proteins is characterized by distinct recognition mechanisms that vary depending on the topology of the G4 structure, including (i) top-stacking interactions, where the protein binds to the exposed surface of the uppermost G-quartet, stabilizing the RG4 structure (Masuzawa and Oyoshi 2020, Zheng *et al.* 2022), (ii) groove-binding, involving interactions within the grooves formed by the loops between G-quartets (Vasilyev *et al.* 2015, Masuzawa and Oyoshi 2020), and (iii) loop-binding, targeting the protruding loop nucleotides that connect the stacked G-quartets (Meier-Stephenson 2022, Ngo *et al.* 2024). The analysis of known G4BPs reveals that these proteins are characterized by highly conserved domains and/or motifs, such as the Arg-Gly-Gly (RGG) domain, also known as the RGG/RG motif or Gly-Arg-rich (GAR) domain (Adlhart *et al.* 2025). The computationally identified “Novel Interesting Quadruplex Interaction Motif” (NIQI), characterized by repeated stretches of RGG or RG amino acids and sequence disorder-promoting residues, has also been identified (Brázda *et al.* 2014, 2018). Other conserved domains include the RNA recognition motifs (RRM), also known as the RNA binding domain (RBD) or ribonucleoprotein domain (RNP), the Zinc finger (ZnFs) domain (Kharel *et al.* 2020), and the Oligonucleotide/oligosaccharide binding (OB)-fold domain. Nevertheless, additional binding motifs, domains, or sequence features may yet be discovered.

### 1.1 Computational approaches for RG4 identification and analysis

Currently, G4BPs are mainly identified through biochemical methods (i.e. pull-down and affinity chromatography) and/or quantitative techniques (i.e. mass spectrometry and Fluorescence Resonance Energy Transfer) (Lee *et al.* 2019). However, these approaches are time-consuming, costly, and, in some cases, impractical, leaving many unanswered questions regarding G4BPs (Dai *et al.* 2023) and making their identification and classification still challenging. Promising advances have been recently achieved thanks to computational and Artificial Intelligence (AI) methods, possibly opening new avenues for a faster and cheaper option in identifying G4BPs. Up to now, Machine Learning (ML) and Deep Learning (DL) techniques have mainly been employed to localize G4s within genomes (Garant *et al.* 2017, Klimentova *et al.* 2020, Barshai *et al.* 2022, Cagirici *et al.* 2022) or to predict DNA and RNA sequences prone to the formation of G4 structures (Kikin *et al.* 2006; Brázda *et al.* 2019). Some resources have been recently released to support G4-related analyses. For instance, the QUADRatlas database integrates experimental data on RNA-binding protein (RBP) binding sites and interaction networks with computational tools predicting DNA and RNA regions prone to form G4 structures (Bourdon *et al.* 2023). While it represents a valuable platform for exploring potential RG4–protein associations, QUADRatlas itself does not predict RG4-binding proteins. Among the few resources addressing RG4–protein interactions, G4FUNNIES combines experimental and computational approaches to evaluate the RG4-binding propensities of nuclear proteins. However, it was developed specifically from chromatin-associated RNA G4BPs identified in nuclear extracts, and its predictor was trained to distinguish proteins binding to folded versus unfolded G4 RNA structures under specific ionic conditions, rather than providing a general G4BP predictor.

In this context, our study introduces for the first time a DL-based model for the classification of RG4-binding proteins and a residue-level scoring scheme highlighting likely binding subsequences. By capturing key determinants of RG4–protein interactions, G^4^REP provides a generalizable, scalable framework for the systematic discovery of human RG4BPs.

## 2 Materials and methods

### 2.1 Data mining

The datasets employed in this work are composed of two different classes of proteins: RG4BPs (positive sequences) and non-RG4BPs (negative sequences). The positive sequences are known human RG4BPs retrieved both from the literature and from the QUADRatlas dataset (Bourdon *et al.* 2023). Negative sequences were selected from proteins that are highly unlikely to bind G4, based on thorough literature searches, UniProt functional annotation, and known structural characteristics (e.g. extracellular or secreted proteins) (the UniProt Consortium 2023). This choice was made to minimize potential bias in the prediction model by ensuring that the negative set comprises proteins with little or no predisposition for quadruplex binding.

### 2.2 Data processing

Each dataset underwent multiple processing steps before being partitioned into training, validation, and test sets. The first step consisted of the removal of duplicates. The second step, homology reduction, a critical process in preparing input data for a neural network model (Li *et al.* 2023), aimed to further eliminate redundancy, thereby minimizing bias, reducing overfitting risk, and enhancing the ability of the model to generalize to unseen data. To achieve this, two rounds of homology analysis were performed using the Many-against-Many sequence searching (MMseqs2), a specialized tool for large-scale protein and nucleotide sequence clustering (Steinegger and Söding 2018). In the first round, MMseqs2 has been applied to cluster binding and non-binding protein sequences based on a threshold of 90% sequence identity and 50% coverage. Under these criteria, sequences were clustered if they shared at least 90% sequence identity and had 50% or more of their sequence aligned. A representative sequence was then selected from each cluster to eliminate redundancy. In the second round, a more stringent threshold of 20% sequence identity was applied while maintaining the 50% coverage requirement. This step was crucial to address the “twilight zone” issue, a region of low sequence identity (between 20% and 35%) where evolutionary relationships between proteins may not be easily detectable. Despite the low sequence identity, proteins in this range may still share a common ancestor. Sequences longer than 2500 or shorter than 50 amino acids were removed to eliminate outliers that could introduce noise and reduce training stability across encoding strategies. For one-hot encoding (OHE), extremely long sequences substantially increased computational cost and memory usage, while very short sequences provided insufficient signal for effective learning. For ESM-2 embeddings, although the model can technically process variable-length sequences, very short proteins (<50 aa) yield embeddings with limited contextual information, and excessively long sequences (>2500 aa) increase variance across batches, hindering optimization. This filtering step ensured consistent input representations and stable training behavior across all models. To assess biological generalization beyond these constraints, we additionally defined a separate test set including sequences with lengths outside the 50–2500 amino acid range, which was used exclusively for post hoc evaluation.

The clusters resulting from the second round of homology reduction and the cleaning procedure (Fig. S1, available as supplementary data at *Bioinformatics* online) were then used to partition the data into training, validation, and test sets in a supervised manner. Clustering was carried out based on full-length sequences to better preserve sequence similarity relationships, avoiding biases introduced by early truncation. To verify that this step order did not affect dataset composition, we also tested the alternative procedure, i.e. applying length filtering (removing sequences shorter than 50 or longer than 2500 amino acids) prior to homology reduction. The comparison showed that the two approaches produced nearly identical results, with class-specific differences below 1% (0.46% for positives and 0.27% for negatives). Therefore, the adopted workflow (homology reduction followed by cleaning) was retained, as it preserves full-length information during clustering while maintaining equivalent dataset composition.

### 2.3 Data partitioning

The partitioning procedure ensured that sequences with at least 20% identity (i.e. those in the same cluster) were assigned to the same set.

The dataset was divided as follows: (i) the training set, including 80% of the sequences, was used for model training and parameter optimization, (ii) the validation set, consisting of 8% of the sequences, was employed to evaluate model performance and fine-tune hyperparameters during training, and (iii) the test set, containing the remaining sequences, was used to assess the final generalization capability of the models. Finally, the dataset was balanced to contain an equal number of positive and negative sequences, ensuring even class representation during model training and evaluation. Balancing was performed by randomly removing only negative sequences while retaining all positive instances.

### 2.4 Sequence encoding and preparation

Protein sequences were vectorized using two encoding strategies: (i) OHE, where each amino acid is represented as a binary vector with a single high (1) value at the index corresponding to the amino acid and zeros elsewhere and (ii) Evolutionary Scale Modeling 2 (ESM-2)-based encoding (Lin *et al.* 2023), which employs a large pre-trained protein language model to generate high-dimensional, context-aware embeddings for each sequence. To address variability in sequence lengths, padding was applied during preprocessing by appending zeros to shorter sequences to standardize input dimensions.

### 2.5 Models architectures

In all model variants, sequences are preprocessed and vectorized before being passed to DL architecture primarily composed of Long Short-Term Memory (LSTM) layers. The architecture of the five proposed DL models can be schematized as follows:

1. Model 1: two LSTM layers followed by two fully connected (FC) layers with a sigmoid activation function and mean pooling.
2. Model 2: two LSTM layers, with an attention layer in between, followed by two FC layers with a sigmoid activation function and mean pooling.
3. Model 3: one CNN layer with Rectified Linear Unit (ReLU) activation function followed by two LSTM layers further followed by two FC layers with sigmoid activation function and mean pooling.
4. Model 4: one CNN layer with ReLU activation function followed by two LSTM layers, with an attention layer in between, further followed by two FC layers with sigmoid activation function and mean pooling.
5. Model 5: one CNN layer with ReLU activation, followed by two FC layers with sigmoid activation and mean pooling.

In Model 1, the input is processed through two stacked LSTM layers, allowing the model to learn dependencies across sequential amino acids. Model 2 and Model 4 introduce an additive attention mechanism (Bahdanau *et al.* 2015) between the LSTM layers, which enables the network to focus on informative parts of the sequence when learning the representation. In Model 3 and Model 4, a Convolutional Neural Network (CNN) layer precedes the LSTM layers. These CNN layers help extract local sequence features prior to recurrent processing. Fully connected layers follow the LSTM (and attention) components, with sigmoid activation to yield outputs between 0 and 1. The mean pooling layer is used after the second FC layer to aggregate results across instances. For implementation details, such as the mathematical formulation of the LSTM updates, attention scoring, and convolutional operations, see the Methods, available as supplementary data at *Bioinformatics* online. Training progress was monitored using early stopping to prevent overfitting, and in cases where early stopping did not yield satisfactory convergence, the maximum number of epochs was conservatively preset to ensure convergence. Model performance was evaluated using standard binary classification metrics. Confusion matrices were used to calculate true positives, true negatives, false positives, and false negatives. From these, we derived accuracy and calculated the area under the ROC curve (AUROC) to assess the model’s ability to distinguish between binding and non-binding proteins.

### 2.6 Models training

All experiments were conducted using Python and PyTorch, with training performed on an NVIDIA^®^ GeForce RTX 4070 GPU. In all the different proposed DL models, the loss function chosen to monitor the model prediction performance is the Binary Cross Entropy (BCE) loss, mainly used in binary classification problems, measuring the prediction performance of a model whose output is a probability value between 0 and 1. To update and optimize all network parameters based on the BCE loss, the Adam optimizer (Kingma *et al.* 2014) (Adaptive Moment Estimation) was used with a fixed adaptive learning rate of 10–5 that allows control of weight adjustments in response to the estimated error during training (Methods, available as supplementary data at *Bioinformatics* online). All models were trained with a batch size of 16. Training progress was monitored using early stopping to prevent overfitting, and in cases where early stopping did not yield satisfactory convergence, the training duration was manually adjusted. Depending on the model architecture, the number of epochs varied between 250 and 1400 for the OHE models and between 200 and 400 for the ESM-2–based models.

### 2.7 Additional datasets: human proteome and stress granules

The human proteome (*n* = 20 435 canonical protein sequences) was obtained from the UniProt reference proteome (the UniProt Consortium 2023). Sequences were filtered to retain only canonical entries. The curated dataset of 473 SG-associated proteins was assembled from the RNA Granule Database (Millar *et al.* 2023).

### 2.8 Structural modeling

AlphaFold3 (AF3) was used with default parameters to model the FAM98B and RG4 complex. The input included the intrinsically disordered C-terminal region of human FAM98B (residues 350–410; UniProt Q52LJ0) and a well-characterized G4-forming RNA sequence (PDB 5DEA (Vasilyev *et al.* 2015). Potassium ions (K^+^), essential for stabilizing G4 structures (Nishio *et al.* 2021), were included in the modeling to maintain the native G-quadruplex conformation.

### 2.9 G^4^REP server implementation

The server is hosted as a freely accessible and user-friendly web server on Apache 2 at https://schubert.bio.uniroma1.it/g4/. It runs on a high-performance dedicated Linux machine equipped with an RTX 3070 GPU and a 12th Gen Intel^®^ Core™ i9-12900KF processor, ensuring efficiency and continuous availability.

### 2.10 Post-classification analysis of G4-binding regions

To localize potential G4-binding regions along the protein sequence, we introduce a score in the G^4^REP server, aimed at integrating positional and compositional preferences derived from our analyses, incorporating neural network–based disorder profiles and residue-specific compositional biases. First, each residue’s flexibility score is obtained from a neural predictor (Erdős and Dosztányi 2024). Second, we compute a local enrichment factor for the key residues Gly, Ser, Tyr, Phe, and Arg by sliding a window of length n along the sequence and counting their normalized frequency. These two streams merge into a composite function, the G^4^REP Score, defined for each window as:


(1) $$G^{4}R\mathrm{EP}=\Phi (\sum_{i=1}^{n}d_{i},\;\frac{\sum \;j{\in \{G,\;S,\;Y,\;F,\;R\}}^{1_{j}}}{n})$$

where 1j flags whether residue j belongs to the G4-binding set. The operator Φ combines summed disorder and motif density into a single scalar ranging from 0 up to the maximum average disorder. To filter out low-scoring regions, we apply a simple gating function:


(2) F(G, B)= {0, G<T B, G≥T

where G is the *G^4^REP Score*, B is the *G^4^REP Model Binding Score*, and T is an empirically chosen threshold. Only windows where G ≥T pass through for further RG4-binding analysis, producing a focused set of candidate regions defined by both structural flexibility and enriched G-S-Y-F-R content.

## 3 Results

Various data filtering and vectorization techniques together with different DL model architectures have been developed to test how they may affect the training and learning capacity of the models, as well as to test their ability in classifying proteins as RG4-binding or non-binding proteins.

### 3.1 Data setup

**Figure 1:**
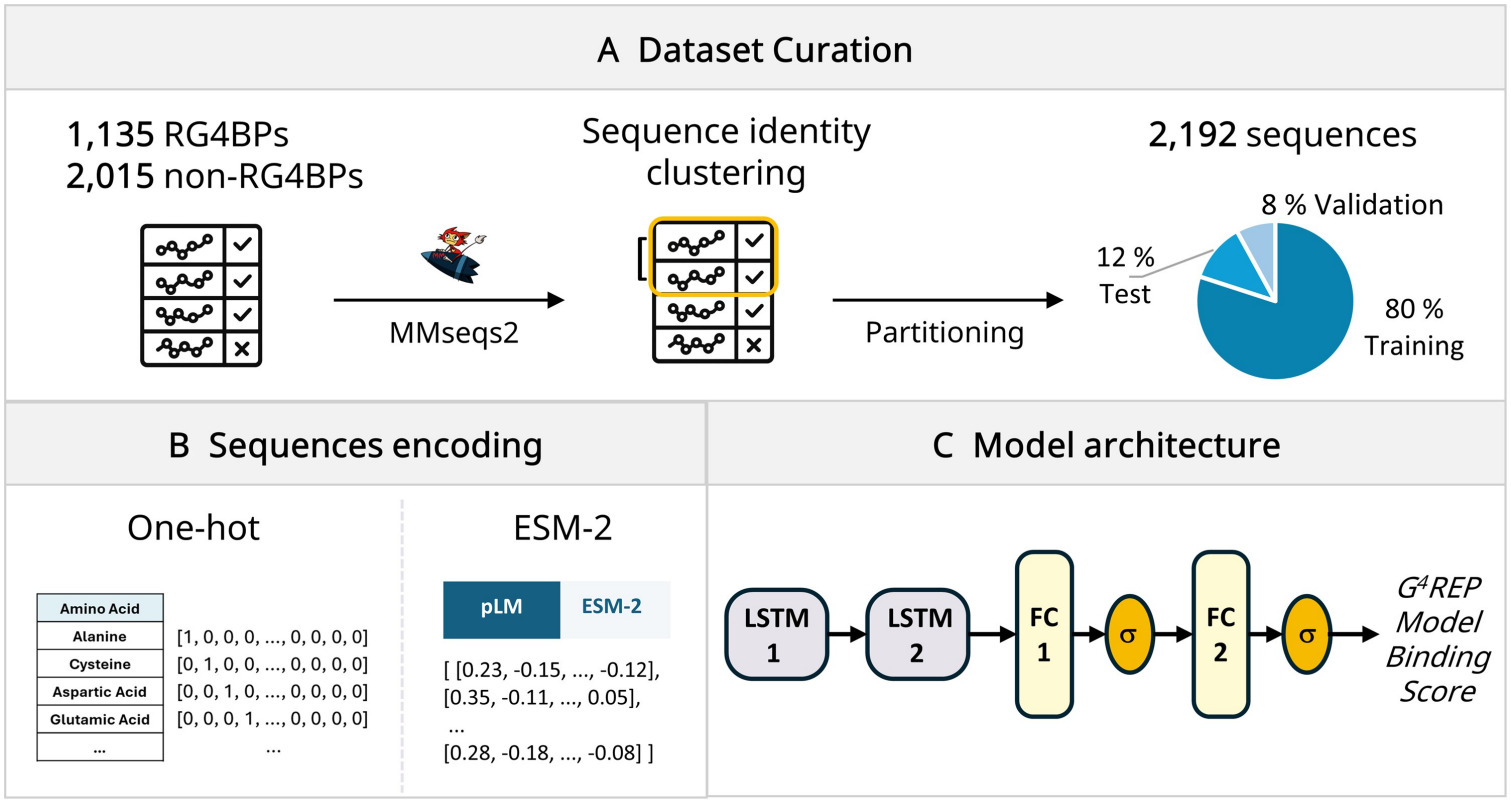
Dataset curation and G4REP Model architecture (A) Dataset curation and preprocessing steps: the dataset includes 1135 RG4-binding proteins (RG4BPs) and 2015 non-RG4BPs. Redundancy was reduced by applying 90% sequence identity filtering and 20% sequence clustering. The dataset was then split into training (80%), validation (8%), and test (12%) subsets. (B) Sequence encoding strategies used for model input: amino acid sequences were encoded using either one-hot encoding or ESM-2 protein language model embeddings (C) *G^4^REP Model* architecture: input sequences are passed through two stacked LSTM layers, followed by two fully connected (FC) layers, each followed by a sigmoid activation function (σ), ending in the *G^4^REP Model Binding Score* indicating RG4BP prediction.

Positive sequences were obtained by merging 240 sequences experimentally identified as RG4-binding with an additional 895 sequences from the QUADRatlas dataset (Bourdon *et al.* 2023), for a total of 1135 sequences. A dataset of 2015 negative sequences was also obtained, as described in the Methods section. Subsequently, all sequences underwent several processing steps: removal of duplicates, homology reduction, and clustering analysis (see Section 2; Fig. 1A). Finally, any sequences longer than 2500 or shorter than 50 amino acids were removed to eliminate excessively long or short sequences that could introduce noise. Additionally, the dataset was balanced to contain equal numbers of positive and negative sequences to prevent learning bias toward the majority (non-binding) class and to improve model stability (see Section 2). In the final dataset, out of a total of 2192 sequences (1096 positives and 1096 negatives), 1748 (874 positives and 874 negatives) were assigned to the training set, 174 (87 positives and 87 negatives) to the validation set, and 270 (135 positives and 135 negatives) to the test set. The labeled datasets are available in File 1, available as supplementary data at *Bioinformatics* online.

### 3.2 Deep learning models evaluation

Protein sequences were encoded using OHE and ESM-2 embeddings, with padding applied as needed (Fig. 1B). Five distinct DL models were implemented, each featuring a different network architecture (see Section 2). The pre-trained esm2_t33_650M_UR50D model (https://github.com/facebookresearch/esm) (Lin *et al.* 2023) containing 33 layers and 650 million parameters, was employed to generate 1280-dimensional embedding vectors for each of the input protein sequences. Notably, although ESM-2 is not explicitly trained on 3D structural data, its embeddings have been shown to encode information that is correlated with protein structure. Through large-scale pretraining on millions of sequences via masked language modeling, ESM-2 captures evolutionary patterns, residue-residue dependencies, and functional motifs that often reflect underlying structural relationships (Zhang *et al.* 2024).

Overall, the results confirmed that models using ESM-2 embedding consistently outperformed those based on OHE. Across all architectures, ESM-2 models achieved strong classification performance on the validation set, with a mean accuracy of 0.83 ± 0.003. Evaluation of Precision, Recall, and F1-score further showed that *Model 1* achieved the most stable results across validation folds and maintained a balanced precision–recall tradeoff (Precision = 0.83 ± 0.02; Recall = 0.84 ± 0.03; F1 = 0.83 ± 0.01; Tables S1 and S2, available as supplementary data at *Bioinformatics* online). The CNN-only model (*Model 5*), which primarily captures local sequence patterns, did not outperform LSTM-based architectures across any of the evaluated metrics. This indicates that modeling longer-range sequence dependencies with recurrent layers provides a measurable advantage for RG4-binding protein prediction. Given that more complex architectures incorporating convolutional or attention layers provided no measurable gain in performance but increased computational cost, we selected the simplest configuration of *Model 1*, hereafter referred to as the *G^4^REP Model*, for downstream analyses, as it provided the best tradeoff between performance consistency, interpretability, and efficiency (Fig. 1C), with a mean accuracy on the test set of 0.84 ± 0.013 and an AUC of 0.917 ± 0.004 (Table S3 and Fig. S2, available as supplementary data at *Bioinformatics* online). To evaluate robustness with respect to sequence length, we further assessed the *G^4^REP Model* on an additional test set including sequences with lengths outside the 50–2500 amino acid range. Performance on this length-extreme test set showed only marginal differences compared to the primary test set, indicating that model predictions are robust to sequence length variability (Table S4, available as supplementary data at *Bioinformatics* online).

### 3.3 Proteome analysis

To both validate the model’s predictive capability and gain new biological insights into RG4BPs, we applied the *G^4^REP Model* to the human proteome (20 435 entries). A small but significant number of proteins (3%, 552) of the whole proteome received a prediction score >0.95, which we define as the threshold for high-likelihood RG4-binding candidates. Given that G4-binding proteins are known to be intrinsically disordered, we examined UniProt disorder annotations to assess whether our predictions aligned with this expected trend. Across the entire human proteome, 55% (11 310) contained at least one disordered region, whereas among proteins predicted with a score >0.95, this proportion increased to 76% (419/552). The enrichment of disorder among high-scoring proteins (Fisher’s Exact Test odds ratio = 2.54 and *P*-value = 5.7 × 10^−23^) underscores the established link between intrinsic disorder and G4-binding propensity (Luige *et al.* 2024). At the same time, the application of the G^4^REP model to the human proteome expands our understanding of G4-binding proteins, uncovering novel, previously unannotated candidates (File 2, available as supplementary data at *Bioinformatics* online). To further explore the relationship between RG4-binding propensity and cellular stress responses, we analyzed stress granule (SG)-associated proteins. A curated SG dataset, consisting of 473 human proteins retrieved from the RNA granule database and classified as “gold standard” (Millar *et al.* 2023) was analyzed using our *G^4^REP Model*, which predicted 99 proteins (21%) as very high-confidence RG4BP (score >0.95). An additional 61% (387 proteins) had a score between 0.5 and 0.95, classifying them as potential RG4BPs with moderate confidence. Consistent with the established link between RG4 binding and intrinsic disorder, 90% (89/99) of the highest-scoring proteins (>0.95) and 83% (189/228) of those with a score >0.9 contained disordered regions, further validating the model’s predictions and reinforcing the key role of disordered regions in SG-associated G4 recognition. To further examine residue-level features that may underpin RG4 recognition and binding specificity, we analyzed the amino acid composition and positional patterns surrounding canonical “RG” and “RGG” motifs in high confidence RG4BPs. Beyond the expected enrichment in Arginine and Glycine, stress granule–associated proteins predicted to bind G4s with very high-confidence (score > 0.95) also show significant enrichment in Asparagine and Glutamine, which are known to interact with guanine residues in both RNA and DNA (Jakubec *et al.* 2016), and in aromatic residues, such as Phenylalanine and Tyrosine (Fig. S3, available as supplementary data at *Bioinformatics* online). Moreover, analysis of amino acid triplets under the same conditions revealed patterns in line with previous observations (Alshareedah *et al.* 2021), such as the enrichment of Proline and Serine adjacent to Glycine, and specific triplets including GGY and GGF (Fig. S4, available as supplementary data at *Bioinformatics* online). The presence of these aromatic residues is consistent with the capping binding mode observed in DNA-G4 structural studies [e.g. PDB: 5CMX (Russo Krauss *et al.* 2016), 2N21 (Heddi *et al.* 2015)] and in prion protein-RNA interactions [PDB: 2RSK (Mashima *et al.* 2013)].

### 3.4 Structural modeling of novel RG4-binding candidates

**Figure 2:**
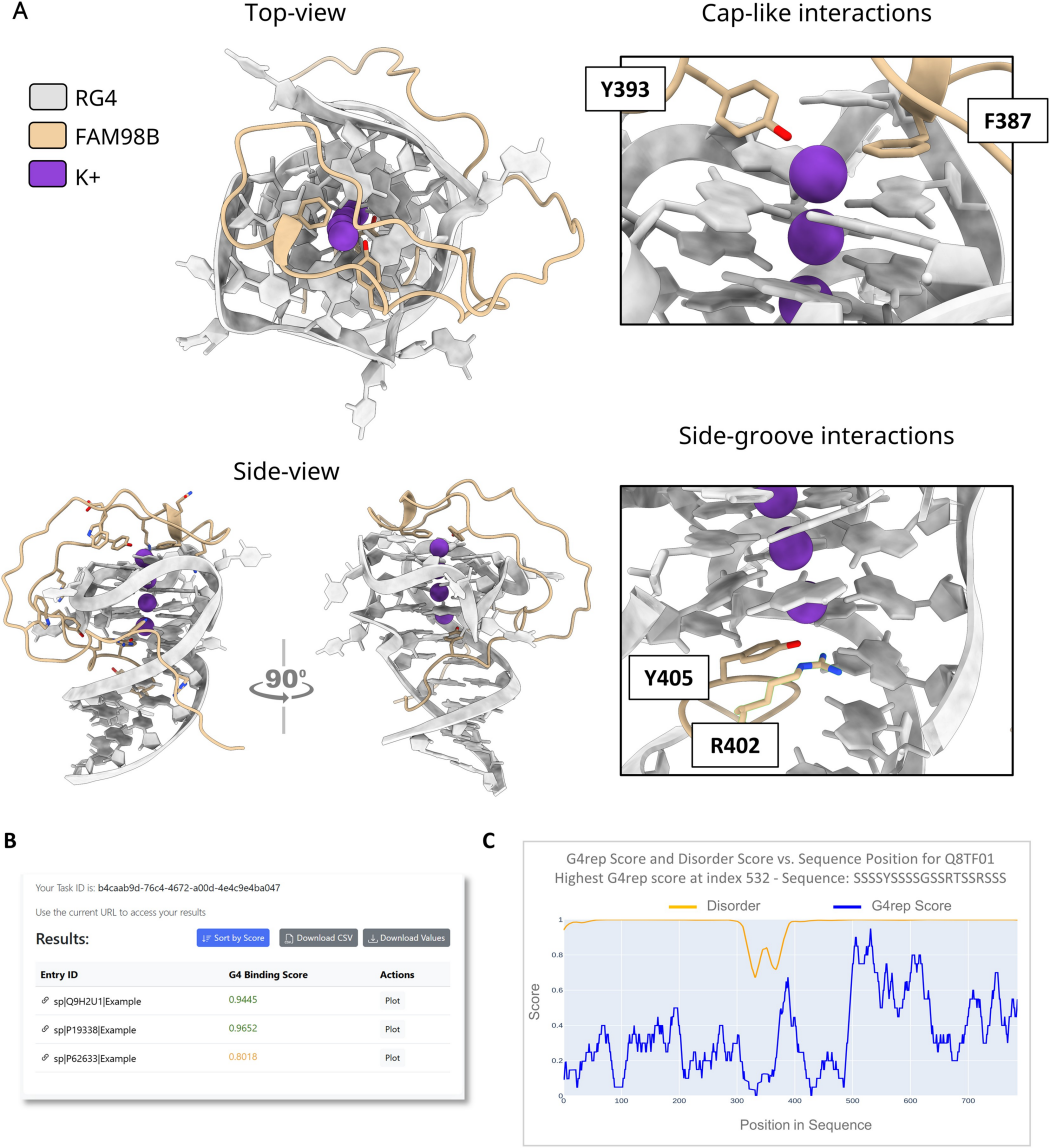
Overview of the compositional analysis and G^4^REP Server (A) Top and side views of the modeled complex between FAM98B (residues 350–410, light yellow) and the RG4 structure (light grey). Representative interactions are shown in sticks, highlighting cap-like contacts over the G-quartet and side-groove interactions involving “RGG” and aromatic residues. (B) View of the *G^4^REP Server* results page: predicted G4 binding scores for input sequences are displayed along with options to view detailed plots or download results. (C) Visualization of per-residue *G^4^REP Score* (blue) and disorder score (yellow) along the sequence of protein Q8TF01.

Among proteins that are predicted as high-confident binders but were not previously annotated, the FAM family of proteins, particularly FAM98A, FAM98B, FAM133A, and FAM133B, emerges as noteworthy candidates. The functional annotations of these proteins remain sparse; however, their involvement in RNA binding and stress response has been identified. For instance, they have been shown to localize to stress granules after various stress stimuli and interact with stress granule-localized proteins such as DDX1, ATXN2, ATXN2L, and NUFIP2, playing a partial role in granules organization (Ozeki *et al.* 2019). By modeling the potential interaction between the FAM98B and RG4 structures with AlphaFold3 (Abramson *et al.* 2024), we observed that the disordered region of FAM98B forms a cap-like interaction over the G4 core (Fig. 2A, top view), with aromatic residues such as phenylalanine and tyrosine positioned above the G-quartet. Along the grooves of the RG4, arginine residues within canonical “RGG” motifs engage in electrostatic and hydrogen bonding interactions with guanine bases, while tyrosine residues participate in stacking-like interactions that stabilize the complex (Fig. 2A, side view). The observed compositional and positional preferences, together with structural modeling, may enhance sequence-based predictions of RG4BP binding modes and provide insights into their underlying interaction mechanisms.

### 3.5 G^4^REP server

The *G^4^REP* server has been developed to provide a user-friendly platform for predicting RG4BPs and analyzing their sequence features to find the most likely subsequence that interacts with the RG4BP. To this end, the *G^4^REP Score* is tailored to identify specific subsequences within predicted RNA G-quadruplex binders that are enriched in intrinsically disordered regions (IDRs) and display characteristic residue biases, as evidenced by the enrichment analysis of the proteome with our DL models. The *G^4^REP Model* is implemented, enabling the analysis of single or multiple protein sequences provided as UniProt IDs, raw sequences, or FASTA files. The output is presented in a tabular format (Fig. 2B), displaying the *G^4^REP Model Binding Score* for each sequence, with the option to visualize a plot of the *G^4^REP Score* alongside disorder propensity (Fig. 2C; see Section 2). As an example, we show the usage of the *G^4^REP Model* and *Server* for the analysis of the Arginine/serine-rich protein PNISR (UniProt: Q8TF01), which has emerged as a previously unannotated RG4BP (*G^4^REP Model Binding Score *= 0.96). The high level of intrinsic disorder may hinder the identification of a specific G4-binding region with respect to its generic RNA binding role (Baltz *et al.* 2012). However, the *G^4^REP Score* can help in pointing to a subsequence in the second low-complexity region of PNISR that exhibits a higher propensity for RG4 interaction (Fig. 2C).

### 3.6 G^4^REP score highlights experimentally defined RG4-binding regions

To further validate the interpretability of the G^4^REP Score, we analyzed well-characterized RG4-binding proteins that were included only in the validation and test sets, and for which experimental data identify the specific regions involved in RG4 interaction. Due to the limited number of proteins with experimentally mapped RG4-interacting subregions, this analysis could only be performed on a few representative cases. For example, FMR1 (Q06787) displays its highest G^4^REP Score within the RGG-box domain (residues 534–548), which is known to mediate G4 binding and for which several complex structures are available (PDB: 2LA5, 5DE5, 5DE8, 5DEA). Similarly, DHX36 (Q9H2U1) shows a maximum score in the N-terminal region (residues 1–51) responsible for G4 recognition and stress-granule recruitment (Lattmann *et al.* 2010). These examples confirm that the G^4^REP Score can successfully highlight experimentally validated RG4-binding regions within independent proteins, demonstrating its biological relevance and interpretability.

## 4 Discussion

RG4s are non-canonical secondary structures formed by guanine-rich RNA sequences that can fold into stable, four-stranded configurations. These motifs have gained increasing attention due to their involvement in key post-transcriptional regulatory processes such as mRNA localization, splicing, stability, and translation. Their functional versatility is further underscored by their enrichment in stress granules and potential roles in cellular stress responses, neurodegeneration, and cancer. However, the proteins that selectively bind RG4s, RNA G-quadruplex-binding proteins (RG4BPs)—remain partly characterized, primarily due to the technical challenges and resource intensity of experimental identification methods. To address this limitation, we developed the *G^4^REP Model*, a DL framework to predict RG4BPs from primary protein sequences. By systematically evaluating five different neural network architectures across multiple encoding techniques, we identified the ESM-2-based LSTM model as the top performer, achieving 84% classification accuracy in distinguishing RG4BPs from non-binders. This underscores the strength of transformer-derived protein embeddings in capturing functional motifs and domain-level information. Although more complex architectures incorporating convolutional and attention layers provided only marginal improvements, the ESM-2 embeddings encode rich biochemical and evolutionary information, whereas the LSTM layers are independently trained to both capture short- and long-range sequential dependencies that underlie RG4-binding specificity.

The application of the model to the human proteome uncovered over 2000 candidate RG4BPs, significantly expanding the known landscape of these regulatory proteins. A substantial fraction of these proteins is localized to the SGs and highly enriched in IDRs and in residues that are often contained in prion-like domains. This outcome not only supports the predictive validity of our model but also reinforces the hypotheses linking prion-like regions to dynamic interactions with G-quadruplexes (Wang *et al.* 2021). Indeed, in addition to the well-characterized “RG” and “RRG” motifs in RG4BPs, it is known that other amino acids—glycine (G), serine (S), tyrosine (Y), arginine (R), and phenylalanine (F)—are enriched between these motifs in polypeptides that undergo RNA-driven phase separation (Alshareedah *et al.* 2021). The notable enrichment of predicted RG4BPs observed among SG-associated proteins is consistent with the growing body of evidence implicating G4s in phase separation processes (Luige *et al.* 2024, Wang *et al.* 2024). Stress granules form under conditions of cellular stress to sequester RNA molecules, and G4 structures may serve as recognition elements that facilitate protein recruitment into these condensates. Within this environment, G4s and their binding proteins may undergo phase separation. This selective compartmentalization could regulate the translation of G4-containing mRNAs, preferentially activating stress-responsive proteins. Overall, our analysis proposes a potential role for RG4 structures in stress granule assembly, aligning with and extending current models of G4-mediated stress adaptation. Future studies will be essential to elucidate the molecular determinants governing these interactions and to assess their broader implications in cellular stress responses and disease.

To complement our sequence-based predictions, we also employed structure-based modeling using AlphaFold3 to explore potential interaction mechanisms between candidate RG4BPs and RG4. Specifically, modeling of FAM98B, a predicted RG4BP, with a representative RG4 structure, revealed a dual binding mode, where “RGG” motifs interact along the duplex/quadruplex boundary grooves via arginine–nucleotide contacts, and aromatic residues such as tyrosine and phenylalanine engage in stacking-like interactions over the G-quartet. These structural features are consistent with known G4 recognition mechanisms and further support the functional relevance of our predictions. Such structural insights offer a mechanistic basis for understanding how IDRs rich in RG and aromatic motifs contribute to G4 recognition, particularly in stress granule environments.

While ESM-2 embeddings incorporate biochemical and evolutionary context, they do not explicitly capture higher-order structural features, such as tertiary and quaternary configurations, which can be crucial for determining RG4-binding specificity. Future improvements could involve integrating structural and biophysical data to enhance the model’s resolution and enable more nuanced predictions, including quantitative assessments of binding strength and mode.

Finally, to make the model easily accessible to the research community, we implemented it as a user-friendly web-based platform. i.e. *G^4^REP Server*, providing an intuitive interface that allows users to analyze protein sequences for G4-binding potential efficiently.

## 5 Conclusions

Here we present a DL framework for predicting RNA G-quadruplex-binding proteins with high accuracy using ESM-2 embeddings and an LSTM architecture. The model reveals new candidate RG4BPs, many linked to stress granules and intrinsic disorder, suggesting functional roles in stress response. The *G^4^REP Server* makes this tool broadly accessible, enabling intuitive exploration of *G^4^REP Model* predictions.

## Supplementary Material

btag088_Supplementary_Data

## Data Availability

The data underlying this article are available at https://schubert.bio.uniroma1.it/g4/
