# Supplementary material for: A deep learning framework for comprehensive prediction of human RNA G-quadruplex-binding proteins
Source: Bioinformatics. 2026 Feb 19;42(3):btag088. doi: 10.1093/bioinformatics/btag088 (PMC13169518; doi:10.1093/bioinformatics/btag088)
Supplement: btag088_Supplementary_Data [file btag088_supplementary_data.zip › SupplementaryFile.pdf]

A Deep Learning Framework for Comprehensive Prediction of Human RNA G-Quadruplex-Binding Proteins

Serena Rosignoli[1*#], Sophie Taraglio[2#], Francesco Di Luzio[3], Elisa Lustrino[2], Dario Marzella[4], Arne Elofsson[5], Massimo Panella[3], and Alessandro Paiardini[2*]

[1]Centre for Regenerative Medicine "Stefano Ferrari", Department of Life Sciences, University of Modena and Reggio Emilia, 41125 Modena, Italy; [2]Department of Biochemical Sciences "A. Rossi Fanelli", "Sapienza" University of Rome, P.le Aldo Moro 5, 00185, Rome, Italy; [3]Department of Information Engineering, Electronics and Telecommunications, "Sapienza" University of Rome, Via Eudossiana 18, 00184 Rome, Italy; [4]Medical BioSciences Department, Radboud University Medical Center, Nijmegen, Netherlands; [5]Department of Biochemistry and Biophysics and Science for Life Laboratory, Stockholm University, 171 21 Solna, Sweden.

S1. Supplementary Methods

This section describes the neural network architectures and computational steps used in our models, including LSTM layers, attention mechanisms, and CNN layers, loss and optimization functions. In Model 1 the output of the preprocessing and vectorization steps has been fed to a network based on two LSTM layers to examine the series of adjacent amino acids and their relationship. Being x^i the i-th encoded amino acid sequence feeding the neural network model, at step h, the first LSTM layer (LSTM layer 1) receives the input vector x^i_h and updates the state of the layer on the base of the computation of the previous inputs of amino acid sequences x^i_{h-1}, x^i_{h-2}, etc.

The output of this layer is computed with the pair of recurrent state equations:

$$c^i_h = f(x^i_h, q^i_{h-1}, c^i_{h-1}, \Theta_c) \quad (1)$$

$$q^i_h = g(x^i_h, q^i_{h-1}, c^i_{h-1}, \Theta_q) \quad (2)$$

where c and q correspond, respectively, to the 'cell' and 'hidden' state vectors of the LSTM layer computed for the current h-th step, $f(\cdot)$ and $g(\cdot)$ are general functions resulting from the combination of the gate equations of the LSTM model, and Θ_c and Θ_q are the layer's weights selected by the training procedure. The output of this layer is the vector q^i, which is a three-dimensional vector of all the hidden states computed for all the different time-steps at the end of the i-th amino acid sequence. The same recurrent system of equations can be used to represent the second LSTM layer (LSTM layer 2). The only difference between these two layers is that the second recurrent layer receives as input the hidden state vector output of the previously described LSTM layer 1. After the second LSTM layer (LSTM layer 2), the obtained output is fed as input to the first fully connected layer, whose output is computed as:

$$d^i = \rho(W_1 k^i + b_1) \quad (3)$$

where k^i is the hidden states vector output of LSTM layer 2, the weight matrix W_1 and the bias vector b_1 are estimated through the learning procedure, and $\rho(\alpha)$ is the activation function (*i.e.*, the sigmoid activation function).

$$L^i = \frac{1}{N}\sum_{i=1}^{N} \rho(W_2 d^i + b_2) \quad (4)$$

where d^i is the output of the first fully connected layer, the weight matrix W_2 and the bias vector b_2 are estimated through the learning procedure, $\rho(\alpha)$ is the activation function (*i.e.*, the sigmoid activation function) and N is the number of instances.
In both FC layers, the sigmoid is employed as activation function to get as output an integer number between 0 and 1 approximated with the Nearest Integer Function:

$$\hat{y}^i = \left[\frac{1}{1+e^{-L^i}}\right] \quad (5)$$

where L^i is the output of the second FC layer after the mean pooling.
In Model 2 and 4, an attention layer also known as *Bahdanau (*Bahdanau et al. 2014*)*, implementing the additive attention mechanism, has been added between the two LSTM layers. The attention mechanism is widely exploited to enhance the capability of neural networks to effectively process and analyse sequential data. It allows to dynamically weigh the importance of different elements in the input sequence when producing an output. In particular, the attention mechanism allows the model to focus on different parts of the input sequence when generating the context vector, which is then fed into the second LSTM layer. Thus, it enhances the representation fed into subsequent layers, enabling a better capture of relevant information. Indeed, the final output of the second LSTM layer would represent the processed output sequence, incorporating the information from the input sequence weighted by attention. In summary, the attention layer receives as input the hidden state output vector from the last time step of LSTM layer 1 and computes, for each time step in the output sequence, a set of attention weights. These weights measure the relevance of each input hidden state to the current output time step. The attention weights are then used to compute a context vector, which is a weighted sum of the hidden states from LSTM layer 1 across all time steps, where the weights are the attention weights. The output vector from the attention layer is then the input for the LSTM layer 2, which can now incorporate the attention-enhanced information. To conclude, the attention mechanism scores each item in the input sequence. The scores are then used to weight the contribution of each item to the output, thus allowing the model to focus on relevant parts of its input data based on the relevance determined by the current state of the model.

In Model 3 and 4, a CNN layer is present before the two LSTM layers. The specific type of CNN layer used, one-dimensional (1D) or two-dimensional (2D), depends on the type of input representation: ESM-2 embeddings or OHE, respectively. Both the 1D and 2D CNNs take as input a four-dimensional tensor and perform the following operations:

$$Y = ReLU(Conv1d(X, W, b)) \quad (6)$$
$$Y = ReLU(Conv2d(X, W, b)) \quad (6')$$

where X is the input feature map (with dimensions: batch size, input channels, sequence length for 1D CNN; batch size, input channels, height, width for 2D CNN), W is the learnable convolutional kernel (with dimensions: output channels, input channels, kernel size for 1D CNN; output channels, input channels, kernel height, kernel width for 2D CNN) and b is the bias term. The convolutional operation convolves the input feature map with the learnable convolutional kernel and adds biases. This operation is defined as:

$$Y_{o,c,i} = \sum_{k=0}^{K-1} \sum_{s=0}^{S-1} X_{o,k,i+s} W_{c,k,s} + b_c \quad (7)$$

$$Y_{o,c,i,j} = \sum_{k=0}^{K-1} \sum_{m=0}^{H_K-1} \sum_{n=0}^{W_K-1} X_{o,k,i+m,j} W_{c,k,m,n} + b_c \quad (7')$$

where Y is the output feature map (with dimensions: batch size, output channels and output length for 1D CNN; batch size, output channels, height, width for 2D CNN), o is the batch index, c is the output channel index, i, j are the spatial indices of the output feature map, K is the number of input channels, k is the input channel index, S is the kernel size (1D), H_k is the kernel height (2D), W_k is the kernel width (2D) and m, n are the spatial indices of the convolutional kernel. Then, the ReLU activation function introduces non-linearity by replacing negative values with zero:

$$Y_{o,c,i} = max(0, Y_{o,c,i}) \quad (8)$$
$$Y_{o,c,i,j} = max(0, Y_{o,c,i,j}) \quad (8')$$

The output of the CNN layer is then flattened and employed to feed the next LSTM layer. The formula of the BCE loss function is described in the following equation:

$$BCE = -\frac{1}{S}\sum_{i=1}^{S} [y_i \log(\hat{y}_i) + (1 - y_i) \log(1-\hat{y}_i)] \quad (9)$$

where S is the number of samples in the training set, y_i is the actual binary label of the i-th sample and $\hat{y}_i$ is the probability estimated by the adopted model that the i-th sample contains that considered type of protein.

S2. Supplementary data

DL models accuracy

Table S1 | Accuracy on the validation set for the five model architectures (Models 1–5) using one-hot encoding (OHE) and ESM-2 embeddings. Values represent mean ± standard deviation.

Model	OHE	ESM-2
**Model 1**	0.806±0.026	0.830±0.007
**Model 2**	0.824±0.018	0.832±0.006
**Model 3**	0.813±0.014	0.837±0.004
**Model 4**	0.783±0.013	0.835±0.004
**Model 5**	0.766±0.015	0.809±0.001

Table S2 | Precision, Recall, and F1-score on the Validation Set for the five model architectures (Models 1–5) using one-hot encoding (OHE) and ESM-2 embeddings. Values are reported as mean ± standard deviation.

Model	OHE	ESM-2
**Model 1**	0.795±0.032<br>0.829±0.054<br>0.809±0.026	0.826±0.016<br>0.838±0.027<br>0.830±0.010
**Model 2**	0.841±0.014<br>0.801±0.044<br>0.820±0.022	0.819±0.006<br>0.853±0.008<br>0.838±0.006
**Model 3**	0.793±0.023<br>0.849±0.049<br>0.819±0.017	0.822±0.004<br>0.856±0.007<br>0.840±0.005
**Model 4**	0.820±0.016<br>0.725±0.040<br>0.770±0.018	0.819±0.003<br>0.857±0.007<br>0.839±0.003
**Model 5**	0.815±0.029<br>0.691±0.032<br>0.747±0.019	0.805±0.029<br>0.820±0.073<br>0.819±0.028

Table S3 | Test set accuracy (top row) and AUC (bottom row) for the five model architectures (Models 1–5) evaluated using one-hot encoding (OHE) and ESM-2 embeddings. Metrics are reported as mean ± standard deviation.

Model	OHE	ESM-2
**Model 1**	0.793±0.018<br>0.858±0.022	0.840±0.013<br>0.917±0.004
**Model 2**	0.790±0.012<br>0.857±0.013	0.844±0.013<br>0.921±0.002
**Model 3**	0.806±0.015<br>0.872±0.009	0.842±0.007<br>0.918±0.002
**Model 4**	0.784±0.018	0.841±0.007

	0.847±0.027	0.918±0.002
**Model 5**	0.750±0.022<br>0.842±0.015	0.814±0.002<br>0.888±0.013

Table S4 | Accuracy (top row) and AUROC (bottom row) of the final *G^4REP model* (Model 1) evaluated on an additional test set including protein sequences with lengths outside the 50–2500 amino acid range. Results are shown for one-hot encoding (OHE) and ESM-2 embeddings and are reported as mean ± standard deviation.

**Model**	**OHE**	**ESM-2**
G^4REP Model	0.793±0.019<br>0.859±0.022	0.853±0.013<br>0.919±0.004

Data processing

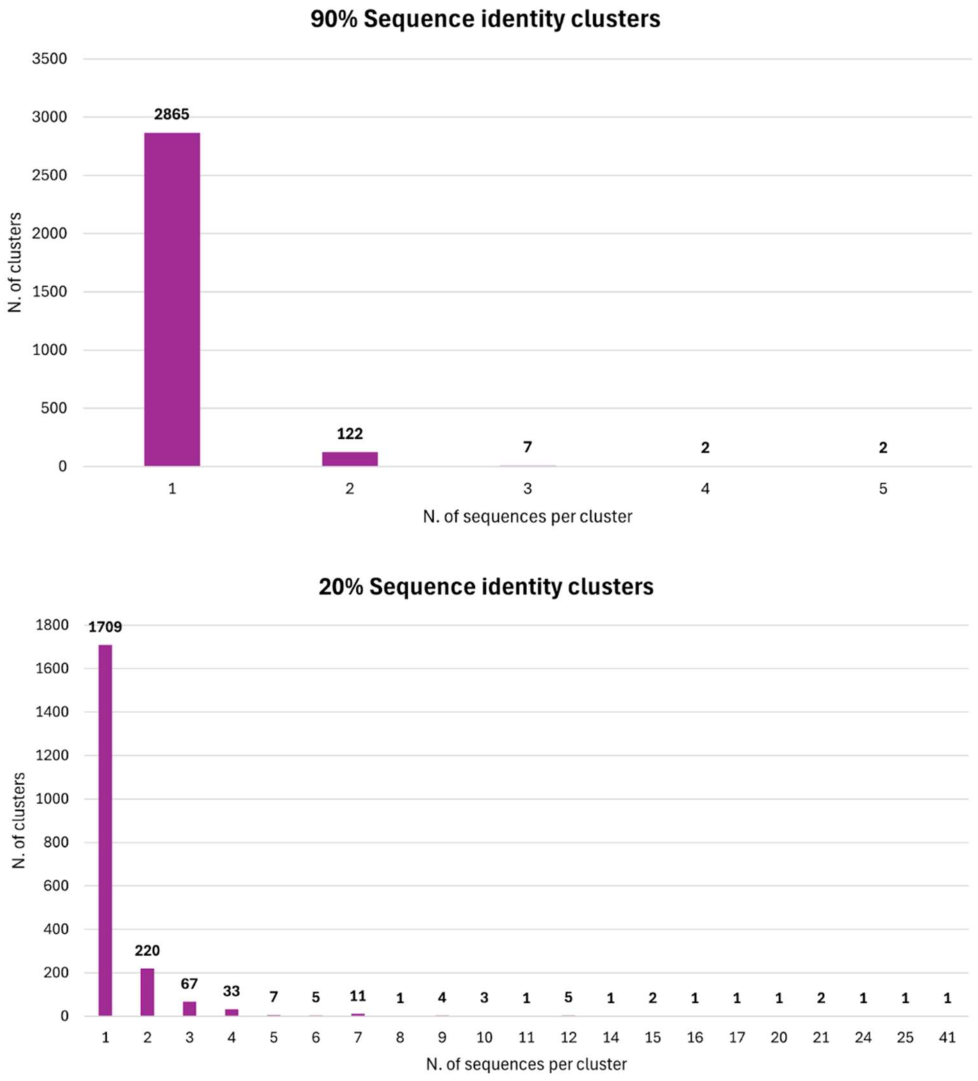

Figure S1. Distribution of sequence identity clusters used for redundancy reduction.
Bar plots showing the number of clusters obtained after (top) 90% and (bottom) 20% sequence identity clustering steps. At 90% identity, the majority of clusters consisted of single sequences (2,865 singletons), confirming effective redundancy removal. Further reduction to 20% identity yielded 2,077 clusters, of which 1,709 were singletons and 220 contained two sequences.

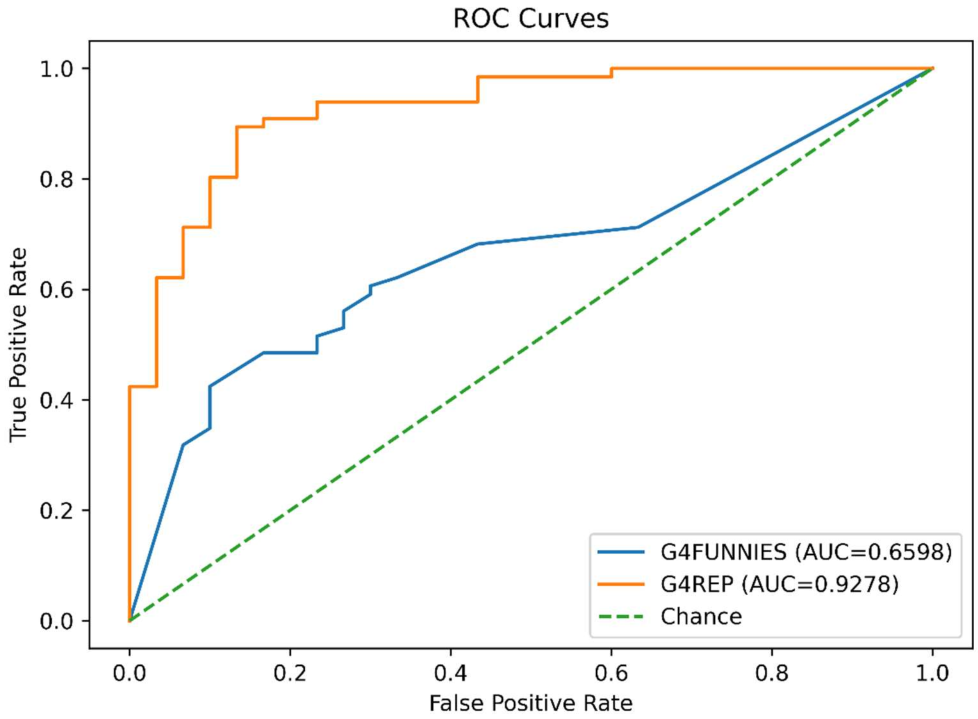

Figure S2. ROC curve comparison between G4REP and G4-FUNNIES.

G4-FUNNIES is the most closely related available tool for RG4-binding protein classification; therefore, we evaluated its performance for comparison. Receiver operating characteristic (ROC) curves showing the performance of G4REP (orange; AUROC = 0.9278) and G4-FUNNIES (blue; AUROC = 0.6598) on the Test set. G4-FUNNIES outputs combined Li^+/K^+ binding propensities rather than a single continuous score; therefore, its predictions were transformed into a continuous ranking scale ranging from 0 (Li^+ 100%, unfolded G4 binding) to 1 (K^+ 100%, folded G4 binding), with intermediate values scaled accordingly. Since G4-FUNNIES did not return predictions for a subset of proteins of the Test set, the corresponding proteins were also excluded from the G4REP evaluation to ensure a fair comparison. This accounts for the slight differences in performance values with respect to those reported in *Supplementary Table S3*.

Amino acid composition analysis

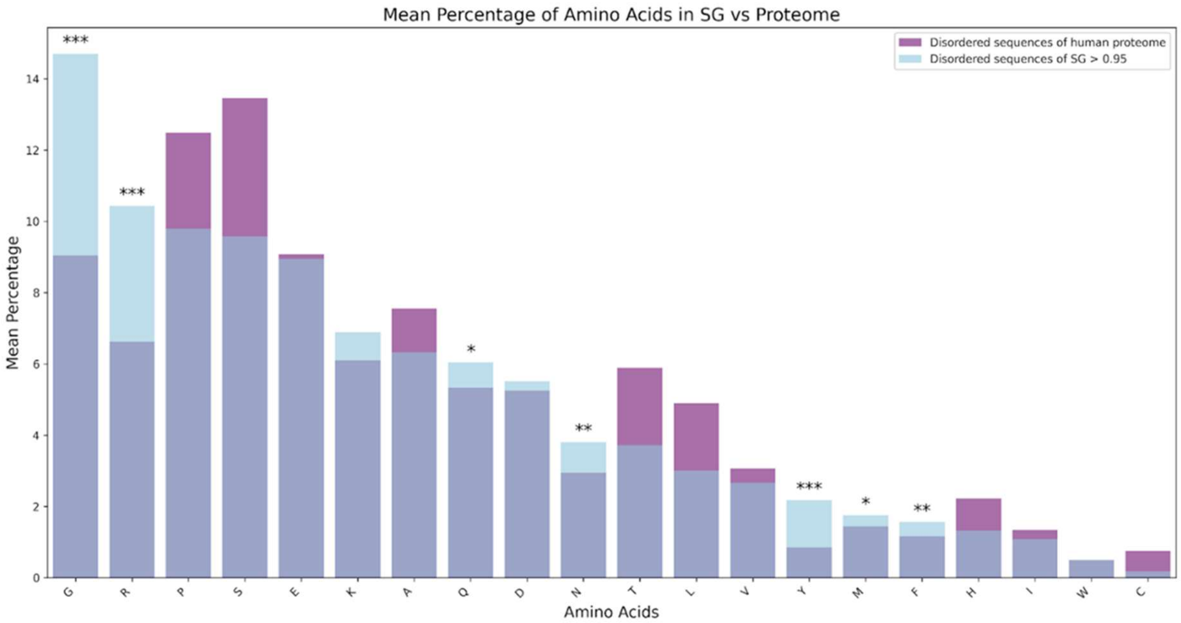

Figure S3. Comparison of mean amino acid composition between disordered regions of the human proteome and disordered regions of SG-associated proteins (prediction score >0.95). For each dataset, the mean percentage of each of the 20 standard amino acids was calculated across all sequences. In the bar plot, light blue bars represent disordered regions of SG-associated proteins, whereas purple bars represent disordered sequences from the human proteome. Statistical significance of amino acid enrichment in SG-associated disordered regions relative to the proteome was evaluated using Welch's t-test for each amino acid. The resulting p-values were corrected for multiple testing using the Benjamini–Hochberg method (False Discovery Rate, FDR). Amino acids with adjusted p-values (p_adj) below 0.05 were considered significantly enriched. Significance levels are indicated with asterisks: p_adj < 0.001 (***), p_adj < 0.01 (**), p_adj < 0.05 (*).

Triplet composition analysis

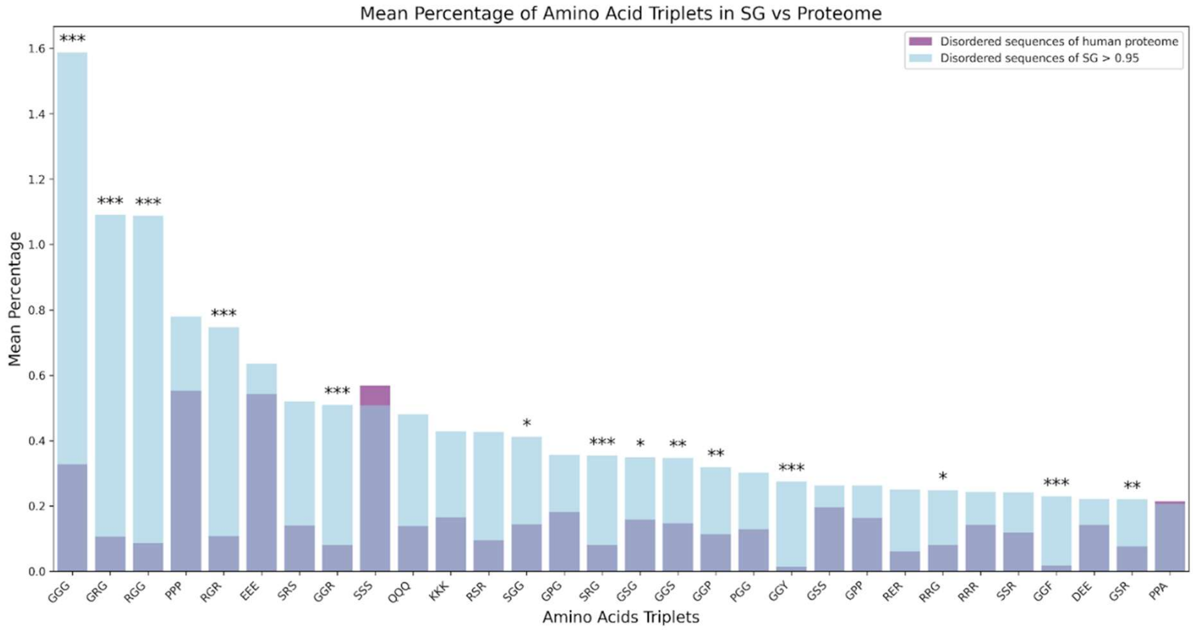

Figure S4. Comparison of mean amino acid triplet composition between disordered regions of the human proteome and disordered regions of SG-associated proteins with prediction scores > 0.95.
For each dataset, the mean percentage of all amino acid triplets was calculated across disordered sequences. In the bar plot, light blue bars represent triplet frequencies in disordered regions of SG-associated proteins (prediction score > 0.95), whereas purple bars correspond to disordered regions of the human proteome. Statistical significance of triplet enrichment in SG-associated disordered regions relative to the proteome was evaluated using Welch's t-test, with p-values corrected for multiple testing via the Benjamini–Hochberg False Discovery Rate (FDR) method. Triplets with adjusted p-values (p_{adj}) below 0.05 were considered significantly enriched. Significance levels are indicated with asterisks: p_adj < 0.001 (***), p_adj < 0.01 (**), p_adj < 0.05 (*).
